# Clinical manifestations of chikungunya among university professors and staff in Santo Domingo, the Dominican Republic

**DOI:** 10.26633/RPSP.2017.64

**Published:** 2017-07-07

**Authors:** Michael A Zingman, Amarilis Then Paulino, Matilde Peguero Payano

**Affiliations:** 1 Columbia University Mailman School of Public Health Department of Epidemiology New York United States of America Department of Epidemiology, Columbia University Mailman School of Public Health, New York, New York, United States of America.; 2 Facultad de Ciencias de Salud Universidad Autónoma de Santo Domingo Santo Domingo Dominican Republic Facultad de Ciencias de Salud, Universidad Autónoma de Santo Domingo, Santo Domingo, Dominican Republic.

**Keywords:** Chikungunya virus, communicable diseases, *Aedes aegypti*, *Aedes albopictus*, the Dominican Republic, Virus chikungunya, enfermedades transmisibles, *Aedes aegypti*, *Aedes albopictus*, República Dominicana

## Abstract

**Objective.:**

To further characterize chikungunya virus infection and its associated clinical manifestations, using a sample of university professors and staff in Santo Domingo, the Dominican Republic.

**Methods.:**

A cross-sectional study with quota sampling by department was performed to obtain a convenience sample of professors (n = 736) and staff (n = 499) at the Universidad Autónoma de Santo Domingo. Surveys were used to collect demographic and infection data during the fall term of 2014. Univariate and bivariate analyses were carried out to quantify infection and clinical manifestation prevalence and to assess relationships of these outcomes with age, sex, and acute phase duration.

**Results.:**

Of 1 236 participants, 49% reported infection (professors = 41%; staff = 61%). Of these, 53% also reported the presence of chronic effects, largely arthralgia (48%). Significant relationships were observed between reported infection and sex (P = 0.023), age (P < 0.001), and occupation (P < 0.001). More headache (P = 0.008) and edema (P < 0.001) in females, more headache (P = 0.005) in younger subjects, and more myalgia (P = 0.006) in those with longer acute symptoms were found. Additionally, more chronic arthralgia (P < 0.001; P = 0.003) and chronic edema (P < 0.001; P = 0.001) in females and older subjects, and more chronic myalgia (P = 0.041) and chronic edema (P = 0.037) in those with longer acute symptoms were observed.

**Conclusions.:**

To the authors knowledge, this is the first population-based chikungunya prevalence study in the Dominican Republic, and the first to explore clinical manifestations in a university setting. The findings reflect results from studies following the 2005 – 2006 Reunion Island outbreak: prevalence of infection and chronic arthralgia, as well as associations with sex, age, and acute intensity. Longitudinal research can provide further insight into these effects.

## RESUMEN

***Objetivo*.:**

Caracterizar de manera más detallada la infección por el virus del chikungunya y sus manifestaciones clínicas, a partir de una muestra de profesores y personal universitario de Santo Domingo (República Dominicana).

***Métodos*.:**

Se realizó un estudio transversal con muestreo por cuotas según los departamentos, a fin de obtener una muestra de conveniencia de profesores (n = 736) y otro personal (n = 499) de la Universidad Autónoma de Santo Domingo. Se recopilaron mediante encuestas los datos demográficos y la información sobre las infecciones durante el semestre de otoño del 2014. Se realizaron análisis univariante y bivariante para cuantificar la prevalencia de las infecciones y de las manifestaciones clínicas, así como para evaluar la relación entre estos resultados y la edad, el sexo y la duración de la fase aguda.

El 49% de los 1 236 participantes indicaron haber padecido la infección (profesores, 41%; otro personal, 61%), de los cuales el 53% también refirieron la presencia de efectos crónicos, principalmente artralgias (48%). Se observaron relaciones significativas entre la infección y el sexo (P = 0,023), la edad (P < 0,001) y la ocupación (P < 0,001). Las mujeres refirieron más cefaleas (P = 0,008) y edemas (P < 0,001); los jóvenes refirieron más cefaleas (P = 0,005); y los sujetos con síntomas agudos de mayor duración refirieron más mialgias (P = 0,006). Además, las mujeres y los sujetos de mayor edad refirieron más artralgias crónicas (P < 0,001; P = 0,003) y edemas crónicos (P < 0,001; P = 0,001), en tanto que los sujetos con síntomas agudos de mayor duración refirieron más mialgias crónicas (P = 0,041) y edemas crónicos (P = 0,037).

**Conclusiones.:**

Hasta donde saben los autores, este es el primer estudio poblacional sobre la prevalencia del chikungunya en la República Dominicana y el primero en el que se exploran las manifestaciones clínicas en un entorno universitario. Los resultados reflejan lo observado en los estudios posteriores al brote del 2005 y el 2006 en la isla Reunión: prevalencia de la infección y artralgias crónicas, así como asociaciones con el sexo, la edad y la intensidad de la fase aguda. Con investigaciones longitudinales se podría arrojar más luz sobre estos efectos.

Chikungunya (CHIK) is a vector-borne disease caused by infection with the chikungunya virus (CHIKV), a ribonucleic acid (RNA) arbovirus from the family *Togaviridae* and genus *Alphavirus* (1, 2). Transmission occurs through two mosquito species, *Aedes aegypti* and *Aedes albopictus* (3, 4). The virus was initially isolated in Tanzania in 1952 and named “chikungunya” after the Makonde word meaning “that which bends up,” referring to symptoms experienced by affected individuals (5).

Infection has occurred mainly in periodic outbreaks in Africa and Asia. In 2006, there were two major epidemics with high attack rates, including over 1 million cases in India and approximately 300 000 cases in Reunion Island, a French territory in the Indian Ocean (3, 6). In December 2013, however, cases of CHIKV infection on the Caribbean island of St. Martin triggered the Pan American Health Organization (PAHO) to release the first alert about CHIKV in the Region of the Americas (2, 7, 8). Since then, local transmission has been confirmed in 43 countries and territories of the Region, with over 1.3 million suspected cases (2).

In February 2014, cases of a febrile syndrome accompanied by arthralgia were reported in the town of San Gregorio de Nigua in the San Cristóbal province of the Dominican Republic. This town is adjacent to a large commercial port that receives daily imports from overseas. These cases, confirmed to be CHIKV infection by the United States Centers for Disease Control and Prevention (CDC; Atlanta, Georgia, United States) in April 2014, marked the beginning of an epidemic in the Dominican Republic. Over 530 000 suspected cases were reported in the Dominican Republic from 2013 – 2014 (7, 9). In 2015, however, newly suspected cases dropped to just 67 (7), a clear decline from the recent peak of the epidemic. Due to the cyclic nature of epidemics and the inability to be re-infected with CHIKV, it is not yet clear if the virus will remain endemic in the country. Therefore, monitoring and surveillance remain crucial, as well as ongoing evaluation of the infestation indexes of *Aedes* mosquitoes (4, 9, 10). According to the national CHIKV preparation and response plan (11), the first entomological studies showed a house index of 0.2 – 2.5 and elevated infestation in water containers used for household consumption.

Prior to symptom onset, CHIKV has both an “extrinsic incubation period” in which it replicates in the salivary glands of the mosquito (average 10 days) and an “intrinsic incubation period” in the human host following the mosquito bite (average 3 – 4 days; range = 2 – 10 days) (3). In the acute phase, symptoms often begin abruptly with high fever and arthralgia (3, 12). In severe cases, joint inflammation can lead to swelling, redness, and limited movement, necessitating rest and absence from daily activities. Initial effects last approximately 3 – 10 days and can also be accompanied by, but not limited to: headache, myalgia, edema, nausea, vomiting, chills, and conjunctivitis (3).

In the majority of cases, the acute phase duration is 2 – 5 days (13). After a sub-acute or convalescent phase of up to 12 weeks in which there is no viremia, chronic effects often follow. Common chronic manifestations include degenerative disc disease, arthritis, depression, anxiety or panic disorders, and visual impairment (14). Continued inflammation can lead to recurrence of arthralgia in affected joints (15, 16). The chronic pain after CHIKV infection can be greater than during the acute disease, resulting in a significant burden on quality of life (17). These effects have been shown to continue for up to 5 years (18). In two studies from Reunion Island, 60% – 63% of patients had persistent arthralgia 1.5 – 3 years after the epidemic, with about half reporting a negative impact on quality of life (17, 19). Several common risk factors for chronic arthralgia have been highlighted, including age > 45 years, intensity of acute pain, and past medical history of arthralgia (20–22).

Much of the current literature has focused on describing barriers and improvements to treatment, control, surveillance, and prevention of CHIKV infection in the Americas (3, 4, 9, 10), but is insufficient in highlighting the impact of infection on specific populations. This paper will attempt to further characterize the recent epidemic in the Dominican Republic and the clinical manifestations of CHIKV infection by observing the specific impact on personnel at the *Universidad Autónoma de Santo Domingo* (Autonomous University of Santo Domingo; Santo Domingo, Dominican Republic UASD). Additionally, it will look at particular factors related to prevalence of clinical symptoms, with an emphasis on chronic effects. Learning more about the behavior of these manifestations may help the medical and public health communities to better develop CHIKV prevention and treatment strategies for the Americas and limit the burden of the chronic phase of infection.

## MATERIALS AND METHODS

A descriptive cross-sectional study was conducted to observe the prevalence, impact, and clinical manifestations of CHIKV infection among professors and staff in a university setting following the outbreak in 2014. The Institutional Review Board at Columbia University Medical Center (New York City, New York, United States) approved all study protocols. Prior to participation, all subjects were informed of the study objectives and scope, and assured that withdrawal would be permitted at any time. All participants provided informed consent prior to the interviews; data were anonymous and confidential.

### Study setting

All research was carried out at UASD, the largest and only public university in the Dominican Republic. Surveys were completed through interviews performed by researchers and students on the main campus in Santo Domingo during the fall term of 2014. Analysis was performed at the Office of Health Sciences Research during the summer term of 2015.

### Sample size and sampling procedure

Two separate sample sizes (professors and staff) were used. The university staff population was 3 348 and the professor population was 2 969. The appropriate probabilistic sample size was calculated using Epi Info™ version 7.1.3 (CDC, Atlanta, Georgia, United States), separately for each group (professors and staff), based on a 95% Confidence Interval (95%CI), 50% expected prevalence (20, 23–29), and 2% design effect. The sample size calculated (plus an additional 10%) was 693 for staff and 748 for professors. Using a quota sampling method by school (professors) or department (staff), selections were made by convenience depending on presence in classrooms, offices, and recreational rooms. In all, the study sample comprised 499 staff and 736 professors.

### Data collection

The interviewers presented themselves to the professors and staff, and described in detail the study’s aims, risks, and benefits, in addition to its informed consent process. Subjects who agreed to participate signed consent forms. Interviews proceeded and survey forms were completed for each participant. Two separate anonymous survey forms were used for data collection, one per group, and later combined for analysis. The information collected was the same for both surveys. Data collection took place over a 2-month period. The surveys were composed of 14 closed- and open-ended questions that collected general data such as age, sex, school, and department, as well as information related to CHIKV infection: clinical manifestations, acute phase duration, diagnosis, treatment, and medical attention. The inclusion criteria were: professors and staff who were active at the time of the survey, were present in the work place when surveys were distributed, and had voluntarily accepted participation in the interviews.

### Data management and analysis

Data were entered into a combined database in Microsoft Excel™ (Microsoft Corp., Redmond, Washington, United States), and statistical analyses were carried out using STATA^®^ version 13 (Stata Corp LP, College Station, Texas, United States). Univariate analyses were performed to observe the variable distribution and to quantify both acute and chronic manifestations from suspected CHIKV infection. Bivariate analyses were performed to explore the potential relationships between CHIKV infection and exposure variables such as age, sex, and length of the acute phase. Separate and combined analyses for professors and staff were performed. Analysis was primarily focused on descriptive statistics and frequency tables, with chi-squared tests performed to assess significance. Due to the cross-sectional nature of the study, prevalence ratios (PR) were used as the effect measure of interest. Age was converted into a three-level categorical variable and acute phase duration was dichotomized around a cut-off point of 9 days. For outcome variables, reported CHIKV infection and clinical manifestations were considered, all of which were binary based on presence. The study specifically investigated arthralgia, headache, myalgia, and edema as these were the most commonly reported chronic effects. The proportion distribution of each effect in the acute and chronic phases was reported.

## RESULTS

The total study population was 1 235 individuals, of which 736 were professors and 499 staff. Of the total, 57% were male, or more specifically, 63% of professors and 47% of staff. The age range was 18 – 84 years (mean 45; standard deviation [SD] = 12.1); among professors, 24 – 84 years (mean 50; SD = 9.8) and staff, 18 – 78 years (mean 38; SD = 11.9) (Table 1).

**TABLE 1.** Demographic characteristics of professors and staff surveyed in a study of chikungunya at a university in Santo Domingo, Dominican Republic, 2014

Variable	Total (*n* = 1 235)		Professors (*n* = 736)		Staff (*n* = 499)	
	No.	%	No.	%	No.	%
Sex						
Female	531	43.00	269	36.55	262	52.51
Male	704	57.00	467	63.45	237	47.49
Age						
Mean, standard deviation	45.26	12.13	49.99	9.82	38.33	11.87
Age category						
< 36 years	281	22.85	42	5.75	239	47.90
36 – 50 years	507	41.22	328	44.87	179	35.87
> 50 years	442	35.93	361	49.38	81	16.23

***Source:*** Prepared by the authors using study data.

Among those reporting suspected CHIKV infection, 54% were male, or more specifically 60% of professors and 48% of staff. This group was 18 – 78 years of age (mean 44; SD = 12.3), with professors having a higher median age of 50 years (SD = 9.6) and staff having a lower median age of 38 years (SD = 11.7). In participants reporting suspected CHIKV infection, 38% were diagnosed by a doctor. Staff reported a higher proportion of diagnoses by a doctor than professors, 50% and 27%, respectively (Table 2).

**TABLE 2.** Demographic characteristics and outcomes of chikungunya virus infection among professors and staff reporting infection at a university in the Dominican Republic, 2014

Variable	Both (*n* = 610)		Professors (*n* = 304)		Staff (*n* = 306)	
	No.	%	No.	%	No.	%
Sex						
Female	282	46.23	123	40.46	159	51.96
Male	328	53.77	181	59.54	147	48.04
Age						
Mean, standard deviation	44.25	12.29	50.38	9.61	38.19	11.65
Age category						
< 36 years	167	27.42	18	5.94	149	48.69
36 – 50 years	237	38.92	128	42.24	109	35.62
> 50 years	205	33.66	157	51.82	48	15.69
Diagnosed by						
Self	358	58.69	207	68.09	151	49.35
Doctor	234	38.36	82	26.97	152	49.67
Neighbor/other	12	1.97	10	3.29	2	0.65
Acute effects						
Arthralgia	567	92.95	275	90.46	292	95.42
Fever	561	91.97	278	91.45	283	92.48
Myalgia	466	76.39	196	64.47	270	88.24
Headache	415	68.03	178	58.55	237	77.45
Rash	362	59.34	154	50.66	208	67.97
Chills	269	44.10	110	36.18	159	51.96
Edema	236	38.69	106	34.87	129	42.16
Retroocular pain	176	28.85	53	17.43	123	40.20
Nausea	136	22.30	39	12.83	97	31.70
Abdominal pain	110	18.03	33	10.86	77	25.16
Bloodshot eyes	81	13.28	25	8.22	56	18.30
Vomiting	61	10.00	16	5.26	45	14.71
Length of time						
< 9 days	450	73.77	221	72.70	229	74.84
≥ 9 days	160	26.23	83	27.30	77	25.16
Chronic effects						
Arthralgia	293	48.03	139	45.72	154	50.33
Edema	49	8.03	34	11.18	15	4.90
Myalgia	40	6.56	15	4.93	25	8.17
Headache	18	2.95	5	1.64	13	4.25
Abdominal pain	11	1.80	4	1.32	7	2.29
Retroocular pain	7	1.15	4	1.32	3	0.98
Fever	6	0.98	4	1.32	2	0.65
Rash	6	0.98	5	1.64	1	0.33
Bloodshot eyes	4	0.66	4	1.32	0	0.00
Nausea	3	0.49	3	0.99	0	0.00
Chills	3	0.49	2	0.66	1	0.33
Vomiting	1	0.16	1	0.33	0	0.00

***Source***: Prepared by the authors using study data.

Of those who completed the survey, 610 (49%) reported suspected infection, including 304 (41%) professors and 306 (61%) staff. The proportion of staff that reported suspected infection by CHIKV was greater than that of professors, and this was statistically significant (*P* < 0.001). It was also observed that the proportion in females was significantly higher than in males, 53% and 47%, respectively (*P* = 0.023). Furthermore, the proportion of younger subjects (< 36 years of age) with CHIKV infection was significantly higher than that of older subjects (> 50 years of age) at 60% versus 46%, respectively (*P* < 0.001) (Table 3).

**TABLE 3.** Proportion of reported CHIKV infection by group, sex, and age category in a study of chikungunya at a university in Santo Domingo, the Dominican Republic, 2014

Variable	Infection (*n* = 610)		No infection (*n* = 623)		P
	No.	%	No.	%	
Overall (*n* = 1 235)	610	49.39	623	50.45	
Staff (*n* = 499)	306	61.32	191	38.28	< 0.001
Professors (*n* = 736)	304	41.30	432	58.70	
Sex					
Female (*n* = 531)	282	53.11	248	46.70	0.023
Male (*n* = 704)	328	46.59	375	53.27	
Age category					
< 36 years (*n* = 280)	167	59.64	113	40.36	0.001
36 – 50 years (*n* = 506)	237	46.84	269	53.16	
> 50 years (*n* = 442)	205	46.38	237	53.62	

***Source:*** Prepared by the authors using study data.

The acute phase duration was reported to be less than 9 days in 74% of participants. The clinical manifestations most reported in the acute phase were (in decreasing order): arthralgia (93%), fever (92%), myalgia (76%), headache (68%), and rash (59%). Staff reported a higher proportion of all of these effects than professors. 53% of participants reported that at least one effect endured past the acute phase (not shown). The manifestations most reported in the chronic phase were (in decreasing order): arthralgia (48%), edema (8%), myalgia (7%), headache (3%) and abdominal pain (2%) (Table 2).

Among the reported acute effects, females were significantly more likely than males to report headache (*P* = 0.008) and edema (*P* < 0.001). A significant relationship between younger age and reported headache (*P* = 0.005) was observed, with a higher proportion in those less than 36 years and 36 – 50 years of age, than among those more than 50 years of age. Further, the authors observed that a longer acute phase duration (9 days or greater) was significantly associated with reported myalgia (*P* = 0.006) (Table 4a).

**TABLE 4a.** Proportion distribution of acute manifestations by sex, age, and acute phase length in a study of chikungunya at a university in Santo Domingo, the Dominican Republic, 2014

Variable	Acute																			
	Arthralgia				P	Headache				P	Myalgia				P	Edema				P
	Yes		No			Yes		No			Yes		No			Yes		No		
	No.	%	No.	%		No.	%	No.	%		No.	%	No.	%		No.	%	No.	%	
Total (*n* = 610)																				
Female (*n* = 282)	267	94.68	15	5.32	0.122	207	73.40	75	26.60	0.008	214	75.89	68	24.11	0.785	141	50.00	141	50.00	< 0.001
Male (*n* = 328)	300	91.46	28	8.54		208	63.41	120	36.59		252	76.83	76	23.17		94	28.66	234	71.34	
> 50 years (*n* = 205)	188	91.71	17	8.29	0.696	126	61.46	79	38.54	0.005	154	75.12	51	24.88	0.301	90	43.90	115	56.10	0.146
36 – 50 years (*n* = 237)	222	93.67	15	6.33		160	67.51	77	32.49		177	74.68	60	25.32		87	36.71	150	63.29	
< 36 years (*n* = 167)	156	93.41	11	6.59		129	77.25	38	22.75		135	80.84	32	19.16		58	34.73	109	65.27	
≥ 9 days (*n* = 160)	145	90.63	15	9.37	0.181	110	68.75	50	31.25	0.821	135	84.38	25	15.62	0.006	72	45.00	88	55.00	0.050
< 9 days (*n* = 450)	422	93.78	28	6.22		305	67.78	145	32.22		331	73.56	119	26.44		163	36.22	287	63.78	

Among the reported chronic effects, the study results show that females were significantly more likely to report arthralgia (*P* < 0.001) and edema (*P* < 0.001) than males. Also, individuals more than 50 years of age were significantly more likely to report chronic arthralgia (*P* = 0.003) and chronic edema (*P* = 0.001) than individuals in both younger age groups. Additionally, a significant relationship was observed between a longer duration of the acute phase and both chronic myalgia (*P* = 0.041) and chronic edema (*P* = 0.037) (Table 4b).

**TABLE 4b.** Proportion distribution of chronic manifestations by sex, age, and acute phase length in a study of chikungunya at a university in Santo Domingo, the Dominican Republic, 2014

Variable	Chronic																			
	Arthralgia				P	Headache				P	Myalgia				P	Edema				P
	Yes		No			Yes		No			Yes		No			Yes		No		
	No.	%	No.	%		No.	%	No.	%		No.	%	No.	%		No.	%	No.	%	
Total (*n* = 610)																				
Female (*n* = 282)	158	56.03	124	43.97	< 0.001	9	3.19	273	96.81	0.745	24	8.51	258	91.49	0.071	39	13.83	243	86.17	< 0.001
Male (*n* = 328)	135	41.16	193	58.84		9	2.74	319	97.26		16	4.88	312	95.12		10	3.05	318	96.95	
> 50 years (*n* = 205)	110	53.66	95	46.34	0.003	8	3.90	197	96.10	0.540	18	8.78	187	91.22	0.291	28	13.66	177	86.34	0.001
36 – 50 years (*n* = 237)	121	51.05	116	48.95		5	2.11	232	97.89		13	5.49	224	94.51		13	5.49	224	94.51	
< 36 years (*n* = 167)	62	37.13	105	62.87		5	2.99	162	97.01		9	5.39	158	94.61		8	4.79	159	95.21	
≥ 9 days (*n* = 160)	87	54.38	73	45.62	0.062	3	1.88	157	98.12	0.349	16	10.00	144	90.00	0.041	19	11.88	141	88.12	0.037
< 9 days (*n* = 450)	206	45.78	244	54.22		15	3.33	435	96.67		24	5.33	426	94.67		30	6.67	420	93.33	

***Source:*** Prepared by the authors using study data.

The results regarding acute effects show that females had a significantly higher (73% vs. 63%) prevalence of headache (PR = 1.16; 95% CI: 1.04 – 1.29) and a significantly higher (50% vs. 29%) prevalence of edema (PR = 1.74; 95% CI: 1.43 – 2.14) than males. Compared to subjects less than 36 years of age, those more than 50 years (PR = 0.80; 95% CI: 0.69 – 0.91) had a significantly lower (61% vs. 77%) prevalence of headache. Those 36 – 50 years of age (PR = 0.87; 95% CI: 0.77 – 0.99) had a significantly lower (68% vs. 77%) prevalence of headache. Moreover, participants with acute phase symptoms of 9 days or greater had a significantly higher (84% vs. 74%) proportion of myalgia (PR = 1.15; 95%CI: 1.04 – 1.26) (Table 4a and Table 5).

**TABLE 5.** Association between clinical manifestations and sex, age and acute phase length, in a study of chikungunya at a university in Santo Domingo, the Dominican Republic, 2014

Variable	Acute						Chronic					
	n	PR^a^	95%CI^b^	n	PR	95%CI	n	PR	95%CI	n	PR	95%CI
Total (*n* = 610)												
Sex	Headache			Edema			Arthralgia			Edema		
Female (*n* = 282)	207	1.16	(1.04, 1.29)	141	1.74	(1.43, 2.14)	158	1.36	(1.15, 1.61)	39	4.54	(2.47, 8.33)
Male (*n* = 328)	208	1.00		94	1.00		135	1.00		10	1.00	
Age category	Headache						Arthralgia			Edema		
> 50 years (*n* = 205)	126	0.80	(0.69, 0.91)	…	…	…	110	1.45	(1.15, 1.81)	28	2.85	(1.40, 5.83)
36 – 50 years (*n* = 237)	160	0.87	(0.77, 0.99)	…	…	…	121	1.38	(1.10, 1.72)	13	1.15	(0.49, 2.70)
< 36 years (*n* = 167)	129	1.00		…	…	…	62	1.00		8	1.00	
Acute phase length	Myalgia						Myalgia			Edema		
≥ 9 days (*n* = 160)	135	1.15	(1.04, 1.26)	…	…	…	16	1.97	(1.02, 3.83)	19	1.89	(1.03, 3.47)
< 9 days (*n* = 450)	331	1.00		…	…	…	24	1.00		30	1.00	

a PR = prevalence rate ratio.

b 95% Confidence Interval.

***Source:*** Prepared by the authors using study data.

Among the reported chronic manifestations, females had a significantly higher (56% vs. 41%) prevalence of chronic arthralgia (PR = 1.36; 95%CI: 1.15 – 1.61) and a significantly higher (14% vs. 3%) prevalence of chronic edema (PR = 4.54; 95%CI: 2.47 – 8.33) than males. In comparison to the subjects less than 36 years of age, those greater than 50 (PR = 1.45; 95%CI: 1.15 – 1.81) had a significantly higher (54% vs. 37%) prevalence of chronic arthralgia and those 36 – 50 years of age (PR = 1.38; 95%CI: 1.10 – 1.72) had a significantly higher (51% vs. 37%) prevalence of chronic arthralgia. Those more than 50 years of age (PR = 2.85, 95%CI: 1.40 – 5.83) also had a significantly higher (14% vs. 5%) prevalence of chronic edema than those less than 36 years. Additionally, participants with acute phase symptoms 9 days or greater had a significantly higher (10% vs. 5%) prevalence of chronic myalgia (PR = 1.97; 95%CI: 1.02 – 3.83) and a significantly higher (12% vs. 7%) prevalence of chronic edema (PR = 1.89; 95%CI: 1.03 – 3.47) (Table 4b and Table 5).

## DISCUSSION

Although the 2014 outbreak in the Dominican Republic appears to have ceased, research must be carried out to better understand the prevalence and risk factors of CHIKV infection and its associated clinical manifestations. This study examines the demographics and clinical profile of university personnel reporting CHIKV infection during the recent epidemic. It is our hope that its findings can promote enhanced prevention, diagnosis, and treatment of CHIKV infection, as well as encourage further population-based prevalence studies of CHIKV in the Region.

The prevalence of reported CHIKV infection in the study sample appears to reflect that of prior epidemics (19, 23–27). The prevalence was estimated to be 35% and 53% in the 2006 outbreaks in La Reunión (Martinique; 19, 23 – 25) and Kerala (India; 26) respectively. This frequent occurrence of infection is likely reflective of the virus infectivity and pathogenicity, as well as the lack of immunity in the Dominican Republic (due to its recent emergence in the Americas).

In participants who reported suspected CHIKV infection, rates of acute clinical manifestations were observed to be comparable to other studies, with some minor differences. As expected, arthralgia and fever were the two most common symptoms (28). This matches the standard case definition, but these symptoms may have been under-reported due to recall bias, especially fever since it tends to be less distinct. The study sample reported myalgia more frequently than the 50% – 60% prevalence reported in various other studies (27, 29). This is a frequently observed symptom after infection with an alphavirus, which has a tropism to muscle cells where it can replicate persistently (6). Additionally, the prevalence of headache was within the typical range (26, 27, 29), while rash was reported at a slightly higher rate than the 40% – 50% prevalence in other studies (12, 26, 27, 30).

We also found that a majority of participants who reported infection experienced at least one symptom that persisted beyond the acute phase. While our survey did not specify a particular timeframe for chronic manifestations, the results are comparable to prior CHIKV outbreaks, for which studies reported that 73.6% and 57.0% of subjects had rheumatic symptoms at 1-month and 15-months post-diagnosis, respectively (26, 31). Since chronic manifestations tend to be more common in individuals with comorbidities and concurrent infections, this could potentially explain the slightly lower prevalence of chronic manifestations reported in our sample of university personnel.

As expected, arthralgia was by far the most common chronic symptom reported (19, 26–29). The high prevalence of chronic arthralgia can likely be explained by adaptive immune responses that promote the persistence of a high CHIKV viral load in joint-associated tissues (32). Patients with persistent arthralgia have been observed to have higher platelet counts and reduced liver enzyme levels than patients with faster recovery (19). The pathogenesis of joint pain from CHIKV infection is not well understood, but research has pointed towards older age, acute pain severity, and presence of comorbid arthralgia as factors promoting chronicity (31).

The high prevalence of chronic arthralgia observed is meaningful due to its reflection of the ongoing disease burden and costs resulting from this large epidemic in Santo Domingo, a major Caribbean city. A recent cohort study in Tolima (Colombia), site of a large outbreak in 2014–2015, found that about half of those with CHIKV infection experienced chronic arthralgia (33). In a smaller cohort study published in a different area of Colombia, the prevalence was even higher. Further research should be conducted in the Dominican Republic and other Latin American countries to determine the impact and timescale of chronic arthralgia among the general population, as well as potential measures to prevent its associated morbidity.

Additionally, the study results revealed a significantly higher prevalence of CHIKV infection in females and in younger subjects. The *Dirección General de Epidemiología* (Directorate General of Epidemiology, Santo Domingo; DIGEPI) reported preliminary case data from the town of San Gregorio de Nigua in March 2014 (34). This data showed a similar proportion of CHIKV infection in females and males, and a peak prevalence in adolescents 15 – 19 years of age. The higher proportion of CHIKV infection observed in females and younger subjects in our study may be linked to increased outdoor exposures. This may be an important finding and demonstrate differences in symptomatic illness among population groups in the Dominican Republic. Alternatively, the finding could be linked to recall bias or inaccurate diagnosis. More research into the demographic variability between outbreaks could provide valuable information.

Finally, in our bivariate analyses, chronic arthralgia and chronic edema were significantly related to female sex and older age. We also observed a significantly higher prevalence of chronic edema and chronic myalgia in those who reported a longer phase of acute symptoms. These findings mirror the results of two retrospective studies following the Reunion Island outbreak (19, 31), which reported that patients with persistent arthralgia or rheumatic symptoms had a higher mean age and were more likely to be female. These studies serve as a valuable point of comparison, but had much smaller sample sizes (56 and 84 patients with persistent rheumatic symptoms, respectively). The aforementioned study in Colombia also found a higher prevalence of chronic arthralgia in females and older individuals (33). A potential explanation for the increased prevalence of symptoms in females is the tendency to have higher rates of headaches, edema, and joint or muscle pain during pregnancy or post-menopause (35). It could also be linked to differences in age between the male and female participants; we found that females in our study were younger [not reported].

The principal strength of our study was the large sample size of 1 236 individuals. Sampling from a large public university population is likely to promote greater generalizability of the findings due to the representative nature of this population. Post-outbreak studies to observe infection and clinical manifestation prevalence in the Dominican Republic and the Americas, as well as risk factors related to poorer outcomes, have not been sufficiently studied to date. This study also investigated reported chronic manifestations, which have been largely unexplored in the CHIKV epidemic in the Americas, and identified factors linked to increased prevalence.

### Limitations

Even after taking into account the methodological criteria applied in the design stage, the results described above should be analyzed carefully, while keeping in mind the study limitations. Similar to most studies of CHIKV infection, past and current symptoms were self-reported without laboratory confirmation, which may have led to over-or under-estimation of the symptom prevalence. The survey was also carried out months after infection in most cases, which could have promoted recall bias and magnification of the symptoms. In Tables 4 and 5, it is also clear that the significance of the results was affected by the sample size in each category. For example, the rarity of chronic headaches made it more difficult for a significant relationship to be observed. Additionally, the surveys would have been more informative if they contained supplementary questions on factors such as socioeconomic status, educational level, recent pregnancy, and concurrent illness. Inclusion of university students in the analysis would also have provided additional information.

### Conclusions

To the authors’ knowledge, this is the first population-based CHIKV infection prevalence study in the Dominican Republic, and the first to explore the prevalence of the clinical manifestations of CHIKV infection in university professors and staff. We observed a high prevalence of CHIKV infection and chronic arthralgia, as well as various significant relationships among key risk factors and clinical manifestations of CHIKV infection. After comparisons were performed, we observed that our findings generally mirror the results of studies from other outbreaks, such as that of Reunion Island.

This research is important in highlighting the impact of CHIKV infection on the Caribbean and the Region of the Americas as a whole, and allowing for improved characterization of resulting clinical manifestations. Additional longitudinal research and population-based prevalence studies could provide further insight into these effects.

## Acknowledgements.

The researchers acknowledge the *Facultad de Ciencias de Salud* (School of Health Sciences) at the *Universidad Autónoma de Santo Domingo* for administrative support, as well as the Columbia University Mailman School of Public Health and its affiliated IFAP Global Health Program (New York, NY) for travel support. We would also like thank the numerous students who conducted the surveys and assisted with initial data collection, as well as all the professors and staff who participated.

## Disclaimer.

Authors hold sole responsibility for the views expressed in the manuscript, which may not necessarily reflect the opinion or policy of the *RPSP/PAJPH* and/or PAHO.

## References

[1] 1. Leparc-Goffart I, Nougairede A, Cassadou S, Prat C, de Lamballerie X. Chikungunya in the Americas. Lancet. 2014;383(9916):514. 10.1016/S0140-6736(14)60185-9 24506907

[2] 2. World Health Organization. Chikungunya - fact sheet. Geneva: WHO; 2015. Available from: www.who.int/mediacentre/factsheets/fs327/en/ Accessed on 13 July 2015.

[3] 3. Moya J, Pimentel R, Puello J. Chikungunya: un reto para los servicios de salud de la Republica Dominicana. Rev Panam Salud Pública 2014;36(5):331-5. 25604103

[4] 4. Weaver SC. Arrival of chikungunya virus in the new world: Prospects for spread and impact on public health. PLoS Negl Trop Dis. 2014;8(6):e2921. 10.1371/journal.pntd.0002921 PMC4072586 24967777

[5] 5. Simon F, Javelle E, Oliver M, Leparc-Goffart I, Marimoutou C. Chikungunya virus infection. Curr Infect Dis Rep. 2011;13(3):218-28. 10.1007/s11908-011-0180-1 PMC3085104 21465340

[6] 6. Assunção-Miranda I, Cruz-Oliveira C, Da Poian AT. Molecular mechanisms involved in the pathogenesis of alphavirus-induced arthritis. BioMed Res Intl. 2013;2013:1-11. 10.1155/2013/973516 PMC3771267 24069610

[7] 7. Pan American Health Organization. Chikungunya: statistics data. Washington DC: PAHO; 2015. Available from: www.paho.org/hq/index.php?option=com_topics&view=readall&cid=5927&Itemid=40931&lang=en Accessed on 20 July 2015.

[8] 8. Pan American Health Organization. Washington, DC: PAHO; 2013. Available from: www.paho.org/hq/index.php?option=com_docman&task=doc_view&gid=23807&Itemid= Accessed on 13 July 2015.

[9] 9. Pimentel R, Skewes-Ramm, Moya J. Chikungunya en la República Dominicana: lecciones aprendidas en los primeros seis meses. Rev Panam Salud Publica. 2014;36(5):336-41. 25604104

[10] 10. Pan American Health Organization. Preparedness and response for chikungunya virus: introduction in the Americas. Washington, DC: PAHO; 2011.

[11] 11. Ministry of Health of the Dominican Republic. Plan de preparación y respuesta frente a brotes de fiebre chikungunya. Santo Domingo: MOH; 2014. Available from: www.paho.org/dor/images/stories/archivos/chikungunya/plan-de-contingencia-chikungunya-version-27-01-2014.pdf?ua=1 Accessed on 12 May 2016.

[12] 12. Borgherini G, Poubeau P, Staikowsky F, Lory M, Le Moullec N, Becquart JP, Outbreak of chikungunya on Reunion Island: early clinical and laboratory features in 157 adult patients. Clin Infect Dis. 2007;44:1401-7. 10.1086/517537 17479933

[13] 13. Ministry of Health of the Dominican Republic, Pan American Health Organization. Taller de revisión de formas severas y atípicas del chikungunya. Santo Domingo: MOH, PAHO; 2014. [Unpublished].

[14] 14. Couturier E, Guillemin F, Mura M, Léon L, Virion J-M, Letort M-J, et al. Impaired quality of life after chikungunya virus infection: a 2-year follow-up study. Rheumatology. 2012;51(7):1315-22. 10.1093/rheumatology/kes015 22427407

[15] 15. Pan American Health Organization. Chikungunya. Washington, DC: PAHO; 2014. Available from: www.paho.org/hq/index.php?option=com_content&view=article&id=9053&Itemid=39843&lang=es Accessed on 13 July 2015.

[16] 16. Bouquillard E, Combe B. A report of 21 cases of rheumatoid arthritis following chikungunya fever. A mean follow-up of two years. Joint Bone Spine. 2009;76(6):654-7. 10.1016/j.jbspin.2009.08.005 19945329

[17] 17. Schilte C, Staikovsky F, Couderc T, Madec Y, Carpentier F, Kassab S, Chikungunya virus-associated long-term arthralgia: a 36-month prospective longitudinal study. PLoS Negl Trop Dis. 2013;7(3):e2137. doi:10.1371/journal.pntd. 0002137. 10.1371/journal.pntd.0002137 PMC3605278 23556021

[18] 18. Yaseen HM, Simon F, Deparis X, Marimoutou C. Estimation of lasting impact of a chikungunya outbreak on Reunion Island. Epidemiol. 2012;S2:003. doi:104172/2161-1165S2-003.

[19] 19. Borgherini G, Poubeau P, Jossaume A, Gouix A, Cotte L, Michault A, et al. Persistent arthralgia associated with chikungunya virus: a study of 88 adult patients on Reunion Island. Clin Infect Dis. 2008;47(4):469-75. 10.1086/590003 18611153

[20] 20. Simon F, Javelle E. Service de pathologie infectieuse et tropicale. Marseille, France: Hôpital d’Instruction des Armées Laveran Marseille; 2007. Available from: www.infectiologie.com/site/medias/JNI/JNI10/CT/JNI2010-Chikungunya_Simon.pdf Accessed on 13 July 2015.

[21] 21. Larrieu S, Pouderouxb N, Pistoneb T, Filleul L, Receveurb MC, Sissoko D, et al. Factors associated with persistence of arthralgia among chikungunya virus-infected travelers: report of 42 French cases. J Clin Virol. 2010;47:85-8. 10.1016/j.jcv.2009.11.014 20004145

[22] 22. Chow A, Her Z, Ong EK, Chen JM, Dimatatac F, Kwek DJ, Persistent arthralgia induced by chikungunya virus infection is associated with interleukin-6 and granulocyte macrophage colony-stimulating factor. J Infect Dis. 2011;203(2): 149-57. 10.1093/infdis/jiq042 PMC3071069 21288813

[23] 23. Soumahoro M, Boelle P, Gauzere B, Atsou K, Pelat C, Lambert B, et al. The chikungunya epidemic on La Reunion Island in 2005 - 2006: a cost-of-illness study. PLoS Negl Trop Dis. 2011;5(6):e1197. doi:10.1371/journal.pntd.0001197. 10.1371/journal.pntd.0001197 PMC3114750 21695162

[24] 24. Roth A, Hoy D, Horwood PF, Ropa B, Hancock T, Guillaumot L, Preparedness for threat of chikungunya in the Pacific. Emerg Infect Dis. 2014;20(8). doi:10.3201/eid2008.130696. 10.3201/eid2008.130696 PMC4111160 25062306

[25] 25. Roth A, Mercier A, Lepers C, Hoy D, Duituturaga S, Benyon E, Concurrent outbreaks of dengue, chikungunya and Zika virus infections – an unprecedented epidemic wave of mosquito-borne viruses in the Pacific 2012-2014. Euro Surveill. 2014;19(41):pii-20929. Available from: www.eurosurveillance.org/ViewArticle.aspx?ArticleId=20929 Accessed on 13 July 2015. 10.2807/1560-7917.es2014.19.41.20929 25345518

[26] 26. Vijayakumar KP, Nair Anish TS, George B, Lawrence T, Muthukkutty SC, Ramachandran R. Clinical profile of chikungunya patients during the epidemic of 2007 in Kerala, India. J Glob Infect Dis. 2011;3(3):221-6. doi:10.4103/0974-777X.83526. 10.4103/0974-777X.83526 PMC3162807 21887052

[27] 27. Pialoux G, Gauzere B, Jauréguiberry S, Strobel M. Chikungunya, an epidemic arbovirosis. Lancet. 2007;7(5):319-27. doi: 10.1016/S1473-3099(07)70107-X. 10.1016/S1473-3099(07)70107-X 17448935

[28] 28. Staples JE, Fischer M. Chikungunya virus in the Americas – what a vectorborne pathogen can do. NEJM. 2014;371(10):887-9. 10.1056/NEJMp1407698 PMC4624217 25184860

[29] 29. Staikowsky F, Talarmin F, Grivard P, Souab A, Schuffenecker I, Le Roux K, Prospective study of chikungunya virus acute infection in the Island of La Réunion during the 2005-2006 outbreak. PLoS ONE. 2009;4(10):e7603. doi:10.1371/journal.pone.0007603. 10.1371/journal.pone.0007603 PMC2764049 19893613

[30] 30. Nhan TX, Claverie A, Roche C, Teissier A, Colleuil M, Baudet JM, et al. Chikungunya virus imported into French Polynesia, 2014. Emerg Infect Dis. 2014;20(10):1773-4. doi:10.3201/eid2010.141060. 10.3201/eid2010.141060 PMC4193187 25271852

[31] 31. Sissoko D, Malvy D, Ezzedine K, Renault P, Moscetti F, Ledrans M, et al. Post-epidemic chikungunya disease on Reunion Island: course of rheumatic manifestations and associated factors over a 15-month period. PLoS Neg Trop Dis. 2009;3(3):e389. doi: 10.1371/journal.pntd.0000389. 10.1371/journal.pntd.0000389 PMC2647734 19274071

[32] 32. Hawman DW, Stoermer KA, Montgomery SA, Pal P, Oko L, Diamond MS, et al. Chronic joint disease caused by persistent chikungunya virus infection is controlled by the adaptive immune response. J Virol. 2014;87:13878-88. doi: 10.1128/JVI.02666-13. 10.1128/JVI.02666-13 PMC3838294 24131709

[33] 33. Rodríguez-Morales AJ, Calvache-Benavides CE, Giraldo-Gómez J, Hurtado-Hurtado N, Yepes-Echeverri MC, García-Loaiza CJ, Post-chikungunya chronic arthralgia: results from a retrospective follow-up study of 131 cases in Tolima, Colombia. Travel Med Infect Dis. 2016;14(1):58-9. doi: 10.1016/j.tmaid.2015.09.001. 10.1016/j.tmaid.2015.09.001 26419952

[34] 34. Dirección General de Epidemiología, Ministerio de Salud Pública de la República Dominicana. Brote de chikungunya: tasa de ataque específica por grupo de edad y sexo, Nigua, San Cristóbal. Santo Domingo: Viceministerio de Salud Colectiva; 2014.

[35] 35. Dunnigan MG, Henderson JB, Hole D, Pelosi AJ. Unexplained swelling symptoms in women (idiopathic oedema) comprise one component of a common polysymptomatic syndrome. Q J Med. 2004;97(11):755-64. doi: 10.1093/qjmed/hch126. 10.1093/qjmed/hch126 15496531

